# Habitat radiomics based on CT for assessing BRCA mutation status in patients with high-grade serous ovarian cancer: a multicenter study

**DOI:** 10.3389/fonc.2026.1784439

**Published:** 2026-02-18

**Authors:** Shuai Zhang, Huayuan Yang, Feng Wang, Haixia Wang, Yuwei Zou, Chengjian Wang, Jinwen Jiao, Xinping Yu

**Affiliations:** 1 Department of Radiology, The Affiliated Hospital of Qingdao University, Qingdao, Shandong, China; 2 Department of Radiology, Qingzhou People’s Hospital, Weifang, Shandong, China; 3 Department of Neurology, Qingzhou People’s Hospital, Weifang, Shandong, China; 4 Department of Pathology, The Affiliated Hospital of Qingdao University, Qingdao, Shandong, China; 5 Department of Gynecology, The Affiliated Hospital of Qingdao University, Qingdao, Shandong, China

**Keywords:** BRCA mutation, CT imaging, habitat radiomics, high-grade serous ovarian cancer, predictive modeling, tumor heterogeneity

## Abstract

**Purpose:**

This study aims to evaluate the potential of CT-based habitat radiomics in predicting BRCA mutation status in patients with high-grade serous ovarian cancer (HGSOC). The goal is to identify radiomic features from distinct tumor habitats that correlate with BRCA mutations and assess the predictive accuracy of various machine learning models.

**Methods:**

A total of 228 patients with histologically confirmed HGSOC were included in this multicenter, retrospective study, with 168 patients in the training cohort and 60 patients in the test cohort. Radiomic features were extracted from the entire tumor and subdivided into five distinct “habitats” based on local tumor features. Predictive models were developed for each of the following: clinical model, radiomics model (based on whole tumor characteristics), five habitat models (habitat1, habitat2, habitat3, habitat4, habitat5), and a combined habitat model (integrating habitat1–5). Five machine learning algorithms (logistic regression (LR), support vector machine (SVM), light gradient boosting machine (LightGBM), extreme gradient boosting (XGBoost)) were applied to each model. The model with the highest average area under the curve (AUC) across the algorithms in the training cohort was selected as the optimal model. Further comparison and evaluation of the optimal models from different algorithms were performed to determine the most reliable one.

**Results:**

Among the five machine learning algorithms, XGBoost showed the highest AUC in the training cohort but exhibited a significant drop in the test cohort, indicating overfitting. In contrast, the SVM model demonstrated more consistent performance across both cohorts, with an AUC of 0.952 in the training cohort and 0.841 in the test cohort, making it the most stable performer among the tested algorithms for predicting BRCA mutation status. Calibration and net benefit analyses further confirmed the potential of the SVM-based habitat model as a non-invasive exploratory tool.

**Conclusion:**

CT-based habitat radiomics offers a promising, non-invasive method for predicting BRCA mutation status in HGSOC. The combined habitat model outperformed traditional clinical and whole-tumor radiomic models by more effectively capturing tumor heterogeneity. SVM, demonstrating stable and reliable performance across datasets, emerged as the most robust model for clinical use. These findings support the integration of habitat radiomics, particularly SVM, for personalized, non-invasive molecular assessment in clinical practice.

## Introduction

High-grade serous ovarian cancer (HGSOC) is the most common and aggressive histologic subtype of ovarian cancer, responsible for 70-80% of ovarian cancer-related deaths (1). The prognosis for HGSOC patients remains poor, primarily due to late-stage diagnosis and chemotherapy resistance (2, 3). Despite initial responses to platinum-based therapies, recurrence rates are high (4), underscoring the need for novel approaches in early detection, prognostication, and personalized treatment strategies.

In recent years, research has focused on the molecular characteristics of HGSOC, particularly BRCA1/2 mutations (5), which impair DNA repair mechanisms, leading to genomic instability and increased sensitivity to platinum-based chemotherapy and poly(ADP-ribose) polymerase (PARP) inhibitors. Consequently, BRCA status plays a pivotal role in therapeutic stratification and the development of personalized management strategies (6–8). However, traditional BRCA mutation testing methods, such as genetic sequencing, are costly, time-consuming, and invasive, highlighting the need for non-invasive alternatives.

CT imaging plays a vital role in the diagnosis and staging of ovarian cancer, offering high-resolution anatomical details essential for detecting ovarian masses, peritoneal implants, and distant metastases (9). However, CT has limitations in assessing the molecular and genetic features of tumors. Radiomics, which involves extracting quantitative features from medical imaging, has emerged as a powerful tool in oncology. It allows for the assessment of tumor heterogeneity, prediction of treatment response, and monitoring of disease progression (10). Some studies suggest that radiomic patterns, such as texture, shape, and intensity distribution, may correlate with genetic alterations (11, 12). However, the utility of CT-based radiomics for predicting BRCA mutation status in HGSOC remains debated. While Yuwei Cao et al (13). demonstrated that a CT-based radiomics nomogram could discriminate BRCA mutation status in HGSOC, Andreas Meier et al. (14) reported that standard CT scans could not accurately assess BRCA status, highlighting the need for improved methods.

Habitat radiomics, a recent evolution in the field, focuses on extracting features from specific tumor regions or “habitats,” which reflect the spatial and functional heterogeneity of tumors. Unlike traditional whole-tumor radiomics, habitat radiomics targets specific areas like necrotic, hypoxic, and proliferative regions within the tumor. This approach has shown potential in identifying molecular characteristics and genetic alterations, offering a more nuanced understanding of tumor biology (15–18).

However, the application of habitat radiomics for predicting BRCA mutation status in HGSOC remains underexplored. This multicenter study aims to evaluate the potential of habitat radiomics based on CT imaging to predict BRCA mutation status in HGSOC patients. We aim to identify radiomic features from different tumor habitats that correlate with BRCA mutations and assess their predictive accuracy. By exploring the relationship between tumor morphology and molecular characteristics, we hope to provide a non-invasive, reliable method for identifying BRCA mutations that could aid in treatment decisions and improve patient outcomes.

## Materials and methods

### Patients

This study represents a multicenter, retrospective analytical investigation aimed at evaluating BRCA mutation status among patients diagnosed with HGSOC. This study was conducted in accordance with the Declaration of Helsinki. The Ethics Committee of the Affiliated Hospital of Qingdao University and Qingzhou People’s Hospital approved this retrospective study and waived informed consent. Patients were enrolled from August 2018 to July 2022. The inclusion criteria specified that participants must have: 1) histologically confirmed HGSOC based on surgical resection; 2) pre-treatment CT images acquired within two weeks prior to the initiation of treatment; 3) available BRCA mutation status; and 4) no prior treatment with chemotherapy, radiotherapy, or targeted therapies prior to imaging acquisition. The exclusion criteria were categorized into technical and clinical factors: 1) technical limitations, including poor contrast enhancement or significant imaging artifacts that precluded high-quality radiomic feature extraction; 2) clinical and biological factors, age < 30 years (as young patients may exhibit distinct tumor biology and clinical presentations); and 3) lesion-specific factors, a maximum tumor diameter of < 2 cm, as small lesions may not provide sufficient volume to reliably capture the spatial heterogeneity required for habitat analysis. After applying these criteria, a total of 228 patients were included in the study, comprising a training cohort of 168 patients from the Affiliated Hospital of Qingdao University and a test cohort of 60 patients from Qingzhou People’s Hospital.

### CT data acquisition

The training cohort was exposed to comprehensive pelvic CT scans using a diverse array of five high-end CT scanners: the Canon AquilionONE, the GE Healthcare Discovery CT750 HD, the GE Optima CT670, the Philips iCT 256, and the Siemens Somatom Definition Flash. The test cohort used two systems: the Philips Brilliance CT 128s and the Siemens Somatom Definition Flash. Notably, significant variations in imaging parameters were observed across scanners, including a range of tube currents from 100 to 300 mA, a consistent fixed voltage of 120 kV, multiplex values spanning 0.599 to 0.984, and a uniform slice thickness of 5 mm. Additionally, the rotation times varied between 0.42 and 0.6 seconds. To enhance image quality, intravenous administration of Iohexol (300 mg iodine/mL) was employed at typical volumes of 85–100 mL, with controlled infusion rates of 2.0–3.0 mL/s. Post-contrast imaging was conducted at 30 seconds (arterial phase), 60 seconds (venous phase), and 90–120 seconds (delayed phase) to capture comprehensive vascular and hemodynamic data.

To address discrepancies in voxel dimensions and window settings across different CT systems, an extensive preprocessing step was implemented. All images underwent re-sampling to a uniform voxel size of 1 × 1 × 1 mm³ using nearest-neighbor interpolation, ensuring preservation of voxel intensity values for subsequent radiomics analysis. Furthermore, standardized window parameters of 350 Hounsfield Unit (HU) (window level) and 35 HU (window width) were applied to ensure consistency across datasets.

### Clinicoradiological risk factors and development of clinical model

Relevant clinical data were collected from hospital information system database, including clinical features such as age, presence or absence of hypertension, presence or absence of diabetes and history of cancer (family history of breast/ovarian cancer or previous history of breast cancer). Tumor-related biomarkers, such as serum cancer antigen 125 (CA125) levels (≤700 U/mL or >700 U/mL) and human epididymis protein 4 (HE4) levels (≤400 pmol/L or >400 pmol/L), were also recorded (19). Imaging characteristics were systematically assessed by a radiologist with >10 years of experience in abdominal imaging. Tumor-related features from CT scans included tumor size (maximum diameter in mm), solid component size (largest dimension in mm), peritoneal disease (PD) pattern (nodular or infiltrative), ascites (presence or absence of ascitic fluid), mesenteric involvement (whether the tumor extended into the mesentery), supradiaphragmatic lymphadenopathy (lymph node involvement above the diaphragm), and bilateral ovarian involvement (whether the tumor was confined to one ovary or involved both ovaries).

Univariate logistic regression was first used to identify clinical and imaging features associated with BRCA status (p < 0.05). Significant features were then included in multivariate logistic regression to identify key predictors of BRCA status (p < 0.05).

### Image segmentation and radiomics features extraction

**Figure 1:**
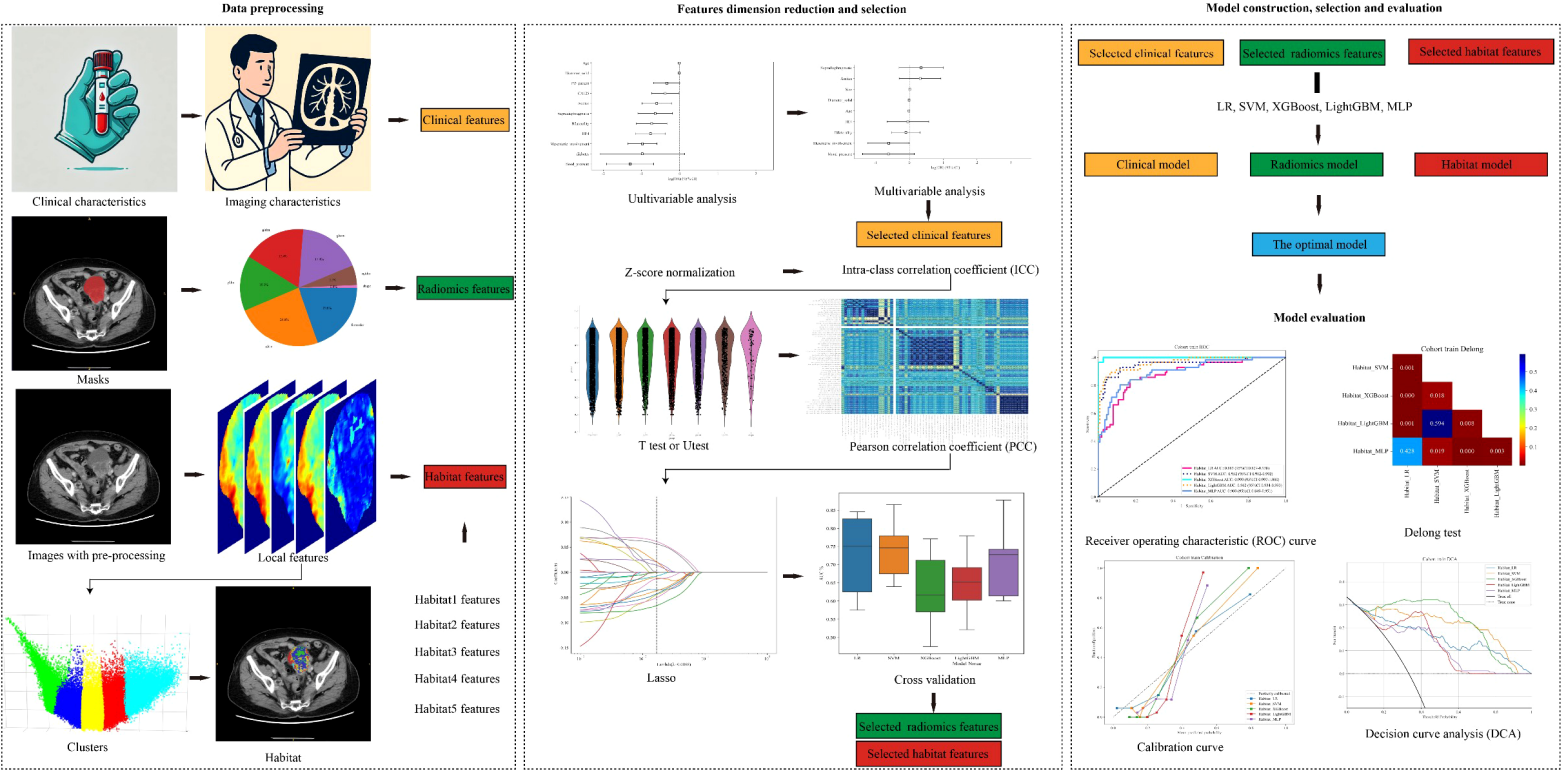
Workflow of the study. Preoperative CT images of patients were retrospectively collected and pre-processed, and then segmented for radiomics features extraction. Radiomics score was constructed after feature selection. Combined model was developed after combing image scores and independent clinical characteristics. Model performance was evaluated in multicenter data by time-dependent ROC curve and KM curve.

The radiomics workflow is shown in **Figure 1**. Tumor regions of interest (ROIs) were manually annotated on axial CT images by two experienced radiologists using ITK-SNAP software (version 3.8.0). Radiomic features were extracted using PyRadiomics (version 3.0) with Python 3.7.12.

A total of 1,834 radiomic features were extracted from the entire tumor, including first-order statistics, shape features, and texture metrics from the Gray Level Co-occurrence Matrix, Gray Level Run Length Matrix, Gray Level Difference Matrix, Gray Level Size Zone Matrix, and Normalized Gray Level Total Density Matrix.

To evaluate spatial heterogeneity, the ROIs were divided into five sub-regions, or “habitats,” using k-means clustering based on local features such as original_firstorder_Entropy, original_glcm_DifferenceEntropy, original_glcm_JointEnergy, original_glcm_JointEntropy, original_ngtdm_Contrast, and Hu values. Radiomic features were then extracted for each habitat, labeled Habitat 1 to Habitat 5.

### Features selection

Radiomic features were standardized using Z-score normalization to adjust for variations in imaging acquisition parameters, ensuring consistency and reproducibility before proceeding with further analysis and model development.

Feature selection was conducted through a multi-step process to identify the most relevant features for model development. First, intra-class correlation coefficients (ICCs) were calculated to assess reproducibility. Thirty CT images were randomly selected, with tumor regions independently segmented by Reader 1 and Reader 2 to evaluate inter-observer variability, and by Reader 1 for intra-observer variability (initial and repeated segmentation after two weeks). Features with ICCs below 0.8 were excluded to ensure high reproducibility. Statistical analysis was performed using a two-tailed u-test, retaining features with a P-value < 0.05. Spearman’s rank correlation coefficient was computed to identify highly correlated features, with pairs showing a correlation > 0.9 being reduced to a single representative feature. Remaining features were further selected using the least absolute shrinkage and selection operator (LASSO) regression, optimized by 10-fold cross-validation and the 1-SE criterion to determine the optimal regularization parameter (λ). Features with nonzero coefficients were retained in the final model.

### Model development and evaluation

We developed predictive models to assess BRCA mutation status using five machine learning algorithms: logistic regression (LR), support vector machine (SVM), light gradient boosting machine (LightGBM), extreme gradient boosting (XGBoost), and multilayer perceptron (MLP). The models were trained and validated on the training cohort and the test cohort. A comprehensive feature set was employed, including selected clinical features, whole-tumor radiomic features, and habitat features (habitat1-5). Additionally, combined habitat radiomic features were incorporated to enhance model performance.

### Further evaluation of the optimal model

The optimal model was selected based on the highest average area under the curve (AUC) across five machine learning algorithms in the training cohort. This multi-algorithmic approach ensures a more robust model selection, as it evaluates performance across different algorithms, providing a balanced and stable assessment of the model’s predictive capabilities.

To validate the optimal model, several advanced evaluation techniques were applied. Receiver operating characteristic (ROC) curves and AUC values were compared for each algorithm (LR, SVM, LightGBM, XGBoost, MLP). Model performance was assessed using metrics like AUC, sensitivity, specificity, positive predictive value, negative predictive value, precision, recall, and accuracy. The DeLong test was used to compare AUC values. Calibration curves evaluated the agreement between predicted and actual outcomes. Finally, decision curve analysis (DCA) assessed clinical utility by evaluating net benefit at various threshold probabilities, highlighting the model’s impact on clinical decision-making and patient outcomes.

### Statistical analysis

The analysis was conducted using SPSS (version 20), custom Python code (version 3.7.12) on Onekey v.4.10.27 platform. Descriptive statistics were employed to summarize continuous and categorical variables, with t-tests and chi-square tests applied for group comparisons. Machine learning models, including LR models, SVM, and MLP, were developed using scikit-learn (version 1.0.2). Advanced gradient boosting models, including LightGBM and XGBoost, were developed using their respective libraries: lightgbm and xgboost.

## Results

### Patient characteristics

The clinical characteristics of patients in the training and test cohorts were presented in **Table 1**. No statistically significant differences were observed between the two groups. Univariate and multivariate logistic regression analyses identified solid size (p=0.041, OR = 1.007, 95% CI = 1.001-1.013) and history of tumor (p<0.01, OR = 11.479, 95% CI = 3.904-33.751) as independent predictors for BRCA status, as detailed in **Table 2**.

**Table 1:** Baseline features of the training and test cohorts.

Features	Training cohort			Test cohort		
	Wild-type BRCA	Muant BRCA	p	Wild-type BRCA	Muant BRCA	p
Age, year	60.68 ± 10.23	57.71 ± 8.20	0.061	57.95 ± 9.88	61.16 ± 10.96	0.386
Size(mm)	121.40 ± 51.05	132.09 ± 59.02	0.223	123.73 ± 57.03	135.74 ± 51.98	0.541
Diameter solid (mm)	54.15 ± 23.85	45.43 ± 24.89	0.026	54.44 ± 24.09	42.84 ± 23.33	0.082
Hypertension			0.089			1
Absent	79(70.54)	47(83.93)		32(78.05)	15(78.95)	
Present	33(29.46)	9(16.07)		9(21.95)	4(21.05)	
Diabetes			0.912			0.795
Absent	104(92.86)	53(94.64)		39(95.12)	17(89.47)	
Present	8(7.14)	3(5.36)		2(4.88)	2(10.53)	
History of tumor			<0.001			0.613
Absent	108(96.43)	41(73.21)		36(87.80)	15(78.95)	
Present	4(3.57)	15(26.79)		5(12.20)	4(21.05)	
PD pattern			0.013			1
Nodular	56(50.00)	16(28.57)		15(36.59)	7(36.84)	
Infiltrative	56(50.00)	40(71.43)		26(63.41)	12(63.16)	
Ascites			0.702			0.704
Absent	61(54.46)	28(50.00)		21(51.22)	8(42.11)	
Present	51(45.54)	28(50.00)		20(48.78)	11(57.89)	
Mesenteric involvement			0.088			0.725
Absent	43(38.39)	30(53.57)		18(43.90)	10(52.63)	
Present	69(61.61)	26(46.43)		23(56.10)	9(47.37)	
Supradiaphragmatic			0.954			0.202
Absent	74(66.07)	36(64.29)		30(73.17)	10(52.63)	
Present	38(33.93)	20(35.71)		11(26.83)	9(47.37)	
Bilaterality			0.75			0.881
Unilateral	60(53.57)	31(55.36)		17(41.46)	9(47.37)	
Bilateral	52(46.43)	25(44.64)		24(58.54)	10(52.63)	
CA125			0.043			1
≤700 U/mL	58(51.79)	19(33.93)		20(48.78)	9(47.37)	
> 700U/mL	54(48.21)	37(66.07)		21(51.22)	10(52.63)	
HE4			0.785			1
≤400 pmol/L	54(48.21)	29(51.79)		24(58.54)	11(57.89)	
> 400pmol/L	58(51.79)	27(48.21)		17(41.46)	8(42.11)	

PD, peritoneal disease; CA125, serum cancer antigen 125; HE4, human epididymis protein 4.

**Table 2:** Univariable and multivariable logistic regression analysis of clinical features.

	Univariate analysis			Multivariate analysis		
Features	OR	95%CI	p	OR	95%CI	p
Age	0.988	0.983-0.992	0	0.985	0.967-1.003	0.167
Size	0.996	0.994-0.998	0.001	1.007	1.001-1.013	0.041
Diameter solid	0.986	0.981-0.991	0	0.986	0.974-0.998	0.059
Hypertension	0.273	0.147-0.506	0.001	0.536	0.249-1.15	0.179
Diabetes	0.375	0.123-1.142	0.147			
Family history of tumor	3.75	1.486-9.459	0.019	11.479	3.904-33.751	0
PD pattern	0.714	0.508-1.004	0.104			
Ascites	0.549	0.373-0.808	0.011	1.368	0.737-2.54	0.405
Mesenteric involvement	0.377	0.258-0.55	0	0.539	0.293-0.994	0.097
Supradiaphragmatic lymphadenopathy	0.526	0.334-0.829	0.02	1.41	0.735-2.707	0.386
Bilaterality	0.481	0.322-0.717	0.003	0.898	0.593-1.358	0.668
CA125	0.685	0.482-0.973	0.076			
HE4	0.466	0.317-0.683	0.001	0.951	0.512-1.766	0.894

OR, odds ratio; CI, confidence interval; PD, peritoneal disease; CA125, serum cancer antigen 125; HE4, human epididymis protein 4.

### Performance of clinical, radiomic, and habitat models: a comparative analysis with emphasis on optimal model selection

**Figure 2:**
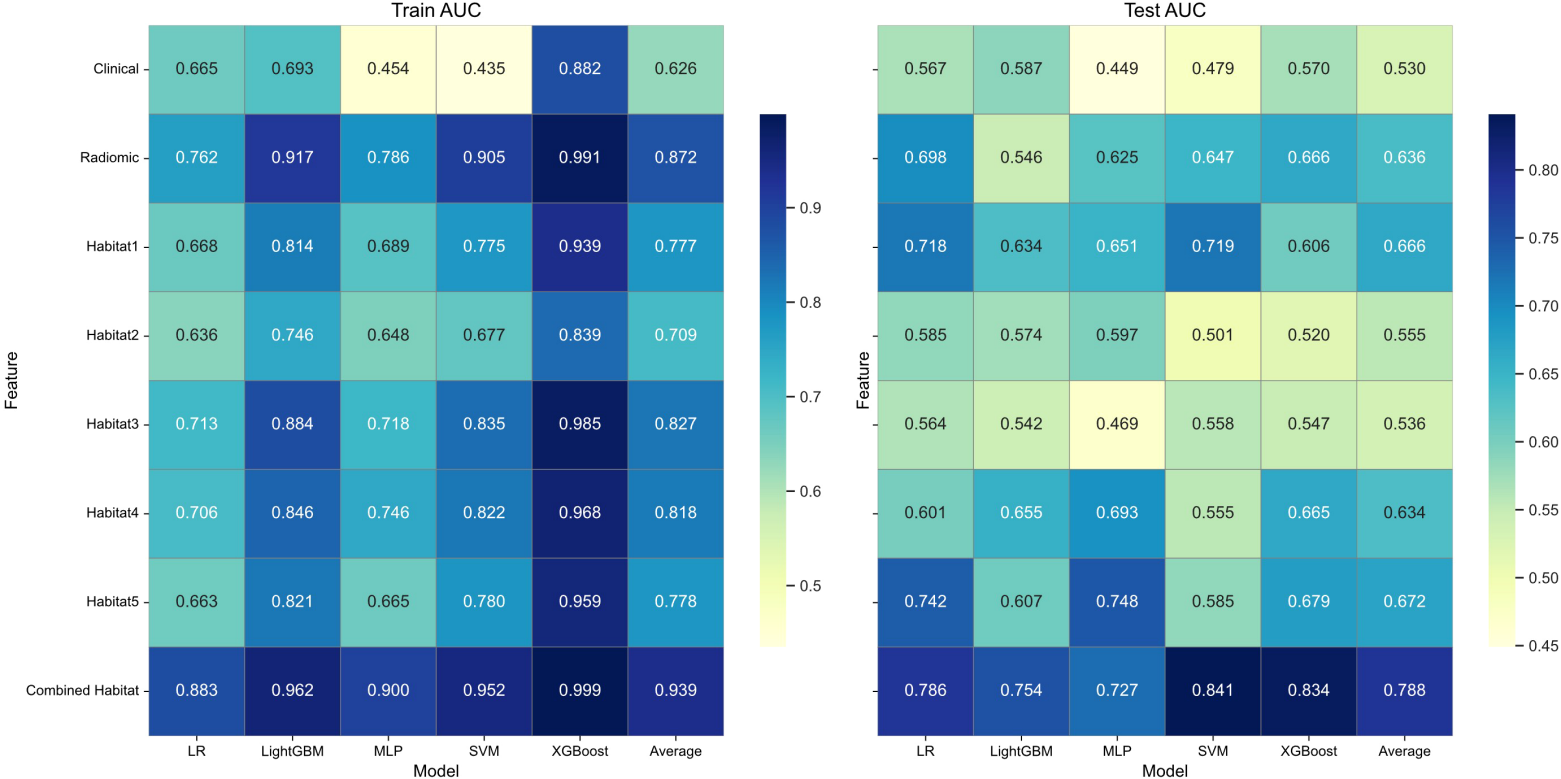
Heatmap illustrating the AUC values for five machine learning algorithms across different models.

**Figure 3:**
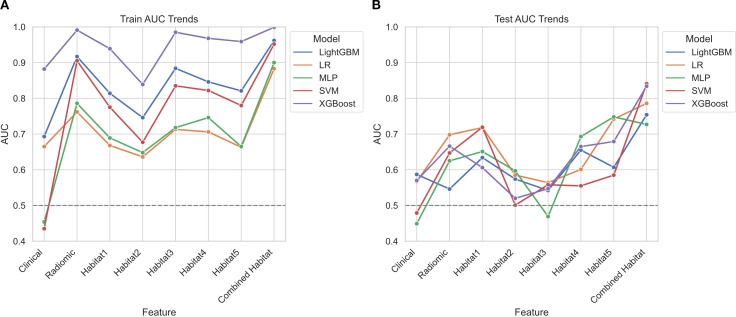
Line chart illustrating the AUC values for five machine learning algorithms across different models. The Line chart displays AUC values for predicting BRCA status in both the training cohort **(A)** and the test cohort **(B)**.

In this study, we conducted an extensive analysis of 40 single-modality models to predict BRCA status, incorporating clinical, radiomic, and different habitats data (habitat1, habitat2, habitat3, habitat4, habitat5, and combined habitat), along with five machine learning classifiers: LR, SVM, LightGBM, XGBoost, and MLP. The heatmap in **Figure 2** displays the AUC values for these models. Owing to its strong performance in training and its sustained AUC in the test cohorts, the combined habitat model was selected as the superior method. It demonstrated the highest predictive stability, surpassing the performance of any single-habitat or whole-tumor radiomic models. (**Table 3**; **Figure 3**).

**Table 3:** Average AUC value across the five machine learning algorithms.

Cohorts	Clinical	Radiomic	Habitat1	Habitat2	Habitat3	Habitat4	Habitat5	Habitat
Training cohorts	0.6258	0.8722	0.777	0.7092	0.827	0.8176	0.7776	0.9392
Test cohorts	0.5304	0.6364	0.6656	0.5554	0.536	0.6338	0.6722	0.7884

### ROC analysis for training and test cohorts

**Figure 4:**
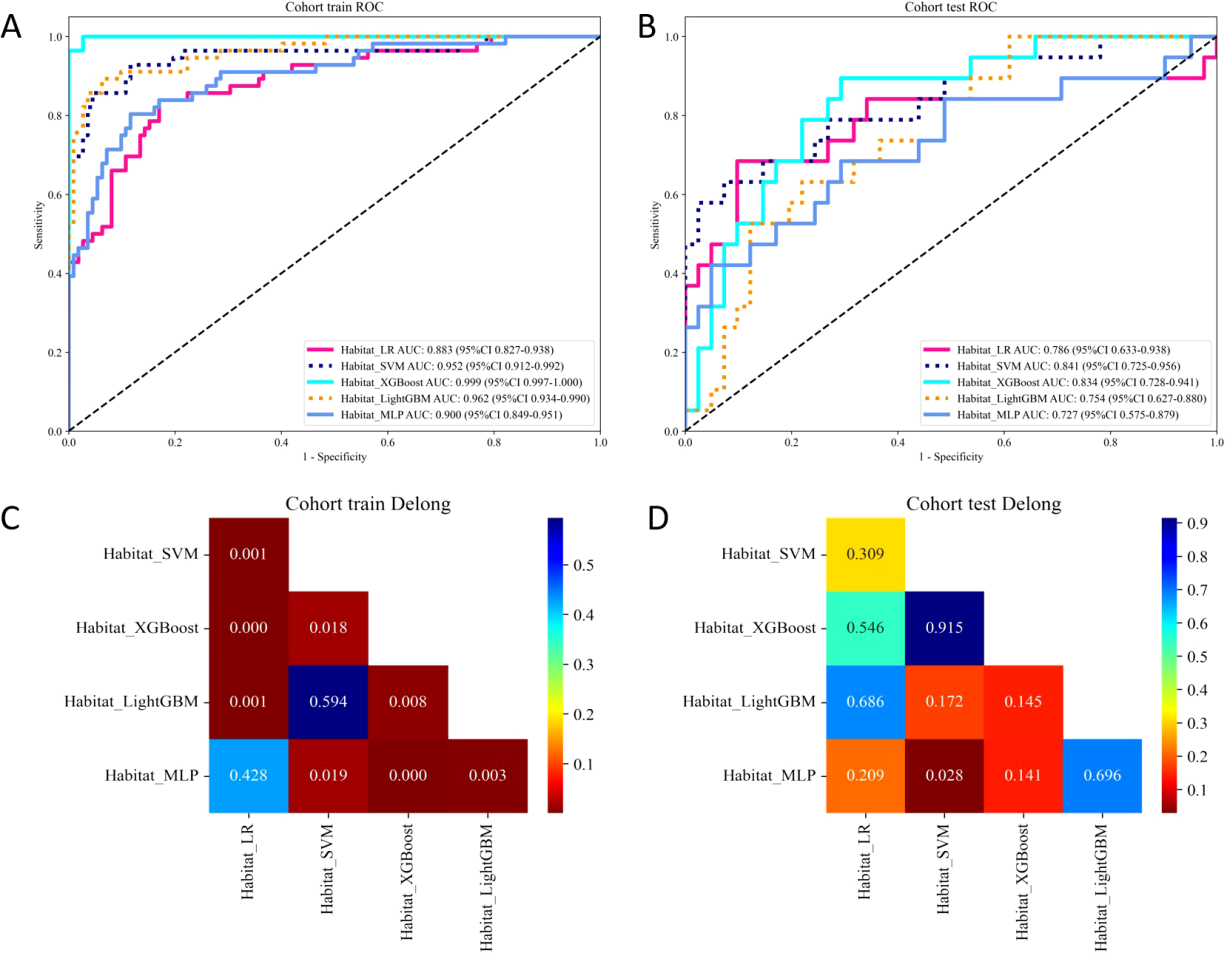
ROC curves of five machine learning algorithms implemented in the combined habitat model for predicting BRCA status in the training **(A)** and test **(B)** cohorts. The Delong test results of the training **(C)** and test **(D)** cohorts.

In the combined habitat model, we evaluated five machine learning algorithms using ROC curves and the DeLong test (**Figure 4**). While XGBoost achieved the highest AUC in the training group, there is a significant drop in its performance in the test group, suggesting possible overfitting. This pattern is also observed in the LightGBM and MLP models, which performed well on the training cohort but showed a decline in the test cohort. In contrast, SVM, while not the top performer in the training cohort, exhibited more consistent performance across both cohorts, with an AUC of 0.952 in training and 0.841 in test. LR also demonstrated relatively stable results but with lower performance compared to SVM. Considering overfitting and generalization, the SVM model appears to be the most reliable for assessing BRCA mutation status, given its balanced performance across both training and test cohorts.

### Calibration and model fit analysis

**Figure 5:**
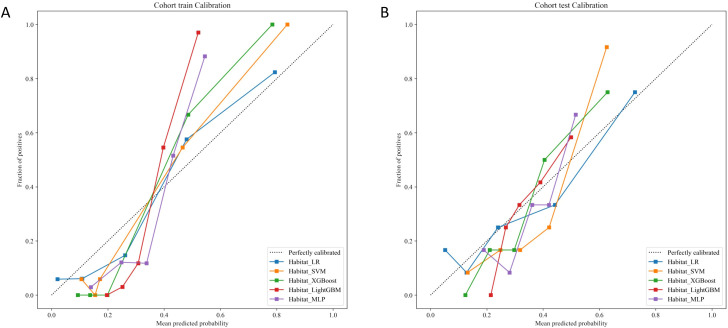
The calibration curves of five machine learning algorithms implemented in the combined habitat model for predicting BRCA status in the training **(A)** and test cohorts **(B)**.

In the combined habitat model, five different machine learning models evaluated on the training and test cohorts, the calibration curves revealed varying degrees of model calibration (**Figure 5**). In the training cohort, all models show relatively good calibration, with LR and SVM displaying the closest alignment to the “perfectly calibrated” line, indicating that their predicted probabilities closely match the observed outcomes. In contrast, LightGBM and MLP appear to show some deviation from the ideal calibration line, suggesting less accurate calibration. For the test cohort, the calibration curves of all five models are similarly distant from the “perfectly calibrated” line, indicating that their calibration performance is more comparable in the test cohort.

### Net benefit analysis

**Figure 6:**
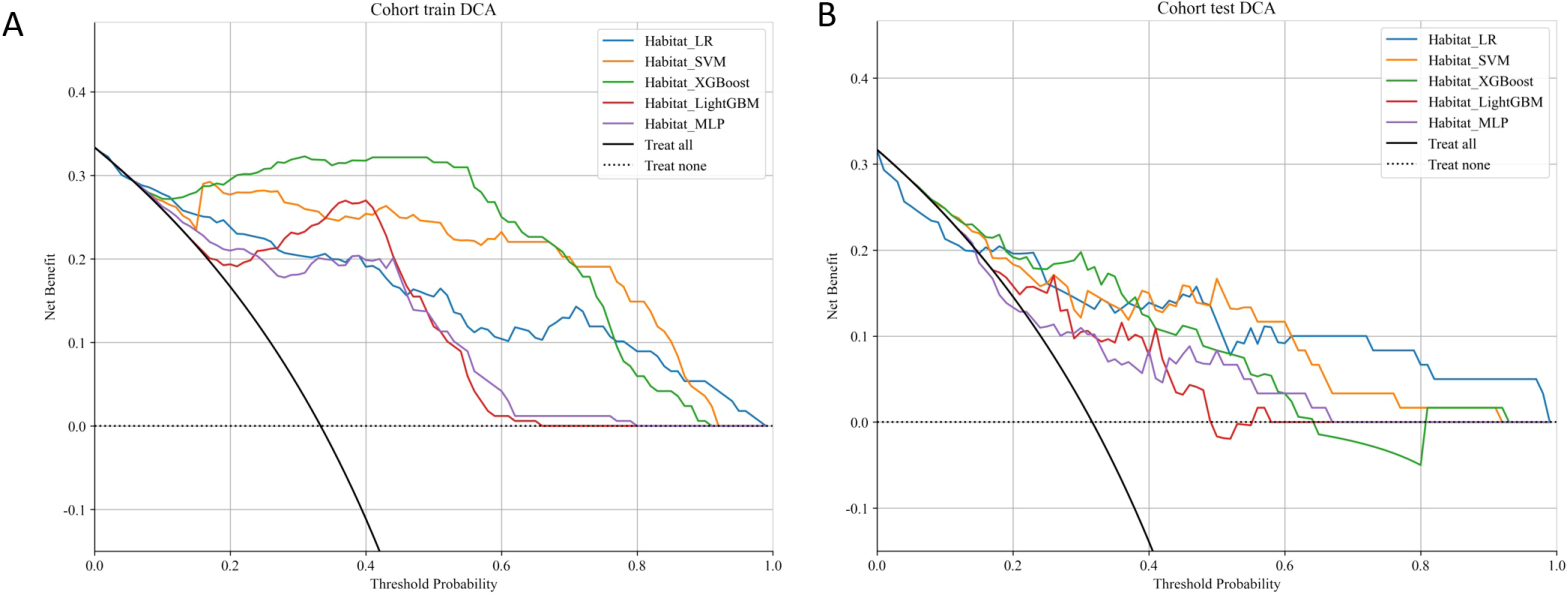
Decision curve analysis of five machine learning algorithms implemented in the combined habitat model for predicting BRCA status in the training **(A)** and test cohorts **(B)**.

We present the DCA performance of five machine learning models in the combined habitat model (**Figure 6**). In the training cohort XGBoost performs best, followed by SVM and LR, while LightGBM and MLP show lower net benefits. In the test cohort, LR and SVM have the highest net benefits, with XGBoost showing reduced performance, suggesting overfitting. Overall, SVM and LR are the most reliable models, offering consistent net benefits in both cohorts, making them the preferred choices for clinical use.

## Discussion

This study aimed to explore the potential of habitat radiomics based on CT imaging for predicting BRCA mutation status in HGSOC patients. Our findings highlight the potential of habitat radiomics as a non-invasive, cost-effective method for assessing BRCA mutations, which play a crucial role in determining treatment response and guiding personalized therapy in ovarian cancer.

Our analysis revealed key insights into the comparative performance of clinical, radiomic, and different habitat-based models. Specifically, the clinical model, which combined clinical and imaging features, achieved an average AUC of 0.5304 in the test cohort across five machine learning algorithms. Clinical features, such as age, hypertension, tumor markers, and imaging characteristics, while important, may not fully capture the molecular complexity of HGSOC, particularly in relation to genetic mutations. Although solid size and tumor history were identified as statistically significant predictors in the multivariate analysis, the overall clinical model’s AUC remained low. This discrepancy suggests that while these clinical factors are associated with BRCA status, their individual and combined predictive power and prevalence are insufficient for reliable clinical screening. Li et al. constructed a model to achieve a comprehensive prediction of BRCA1/2 gene mutations in ovarian cancer by integrating pathological tissue features, cell features, and clinical characteristics, while the model achieved AUC values ranging from 0.429 to 0.698 (20). Consequently, clinical model, as currently constructed, lacks sufficient discriminative power for clinical utility despite the statistical significance of its individual components.

Similarly, the radiomic model, based on whole-tumor ROI features, yielded a higher average AUC of 0.6364 across the five algorithms in the test cohort. The improvement over the clinical model underscores the added value of radiomic analysis in capturing tumor heterogeneity, including texture, shape, and intensity features that might reflect underlying genetic alterations. Research has demonstrated a strong association between radiomic features and underlying pathological alterations, indicating that imaging can noninvasively reflect broader pathological characteristics (10, 21, 22). By interrogating the texture, shape, and signal intensity patterns within tumors, radiomics may unveil insights into the tumor’s genetic landscape. Whole-tumor radiomics has shown promise in capturing subtle tumor characteristics, and it outperformed clinical features alone. However, this model still falls short compared to the habitat model, suggesting that considering the entire tumor as a homogeneous entity in radiomic analysis may not fully leverage the spatial and functional heterogeneity inherent to tumors (18).

In contrast to the clinical and radiomic models, the combined habitat model achieved the highest performance with an average AUC of 0.7884 in the test cohort. The habitat model combines features from different tumor subregions, or “habitats,” which are defined through k-means clustering analysis based on local imaging characteristics. The habitat model aims to capture the heterogeneity and microenvironmental variations within tumors, including hypoxic, necrotic, and proliferative areas, which are often linked to molecular features such as BRCA mutations (11, 23). The biological basis for the superior performance of the combined habitat model lies in its ability to quantify the spatial manifestation of tumor heterogeneity driven by genetic alterations. For instance, radiomic features such as original_firstorder_Entropy and original_glcm_JointEntropy measure the randomness and complexity of voxel intensity distributions. And the GLCM-based features reflect the local variations in cell density and vascularity. The combined habitat model accounts for these differences, enabling the extraction of a more nuanced set of features that better reflect the tumor’s genetic landscape and its response to treatment. Our findings are consistent with recent studies by Wang et al., who demonstrated that radiomics based on habitat could predict the Ki-67 expression accurately and outperformed conventional radiomics model in patients with HGSOC (24).

Interestingly, when we assessed the performance of individual habitat models, the average AUC across the five machine learning algorithms was lower than that of the combined habitat model. This result can be explained by the fact that focusing on a single habitat misses important interactions and relationships between different regions of the tumor. Each individual habitat represents only a small portion of the tumor, and capturing only one region at a time does not provide a complete picture of tumor heterogeneity. Tumor habitats such as necrotic or hypoxic zones may exhibit distinct features from proliferative regions, and ignoring these inter-habitat dynamics reduces the model’s ability to accurately predict genetic alterations like BRCA mutations.

While the combined habitat model demonstrated promising results, the performance of the five different machine learning algorithms varied significantly. Notably, the XGBoost model achieved the highest AUC in the training cohort but showed a significant drop in performance in the test cohort, suggesting overfitting. In contrast, the SVM model, while not the top performer in the training cohort, showed more consistent performance across both the training and test cohorts. With an AUC of 0.952 in the training cohort and 0.841 in the test cohort, SVM demonstrated a good balance between training accuracy and generalization, making it a more reliable choice for predicting BRCA mutation status in patients with HGSOC. Additionally, the LR model also demonstrated relatively stable performance with good calibration. Considering the results from calibration, net benefit analysis, and ROC performance, SVM emerges as the most reliable and robust model for clinical application. Its consistent performance across both cohorts, along with a lower tendency to overfit, makes it the preferred model for clinical use in assessing BRCA mutation status. The LR model, while less powerful than SVM, may still be valuable due to its simplicity and interpretability, especially for clinical settings where model transparency is crucial. The integration of habitat radiomics into clinical practice would require further test and refinement. Future studies should focus on optimizing imaging protocols and standardizing feature extraction methods to ensure reproducibility across different institutions and imaging platforms. Additionally, the application of deep learning techniques could enhance the ability to identify subtle patterns in medical images that are difficult to capture with traditional radiomics approaches. Longitudinal studies would also be beneficial to assess how radiomic features evolve over time in response to treatment and how they correlate with clinical outcomes.

While our study provides compelling evidence for the potential of habitat radiomics in predicting BRCA mutation status, several limitations should be considered. First, the retrospective nature of the study and the variability in CT imaging protocols across different centers may limit the generalizability of our findings. Despite efforts to standardize imaging parameters and voxel sizes, variations in scanner types and acquisition settings could still introduce bias into the analysis. Second, the sample size, particularly the external test cohort, remains relatively modest. Although this is one of the few multicenter studies specifically targeting BRCA status in HGSOC through habitat radiomics, the statistical power may limit the detection of subtle radiomic-genomic correlations. Moreover, while the radiomic features selected were associated with BRCA status, the underlying biological mechanisms linking these features to specific genetic alterations remain unclear. Therefore, our findings should be interpreted as strong preliminary evidence, and further validation in larger, prospectively recruited cohorts with standardized imaging protocols is warranted to confirm the clinical utility of the model.

## Conclusions

In conclusion, CT-based habitat radiomics offers a promising, non-invasive method for predicting BRCA mutation status in HGSOC. The combined habitat model outperformed clinical and radiomic models by capturing intratumoral heterogeneity more effectively. These results support the potential role of habitat radiomics, particularly SVM, as a valuable tool for further exploration in personalized, non-invasive molecular assessment in clinical practice, though its clinical readiness requires further large-scale prospective validation.

## Data Availability

The original contributions presented in the study are included in the article/supplementary material. Further inquiries can be directed to the corresponding author.
